# The Impact of Disulfiram Treatment on the Reinforcing Effects of Cocaine: A Randomized Clinical Trial

**DOI:** 10.1371/journal.pone.0047702

**Published:** 2012-11-08

**Authors:** Colin N. Haile, Richard De La Garza, James J. Mahoney, David A. Nielsen, Thomas R. Kosten, Thomas F. Newton

**Affiliations:** Baylor College of Medicine, Menninger Department of Psychiatry & Behavioral Sciences, Michael E. DeBakey Veterans Affairs Medical Center, Houston, Texas, United States of America; California Pacific Medicial Center Research Institute, United States of America

## Abstract

**Background:**

Clinical trials indicate that disulfiram (250 mg/d) reduces cocaine use, though one study found that treatment with lower doses of disulfiram (62.5 and 125 mg/d) increased cocaine use. We conducted the present study to better understand how disulfiram alters the reinforcing effects of cocaine in cocaine users.

**Methods:**

Seventeen non-treatment seeking, cocaine-dependent volunteers participated in this double-blind, placebo-controlled, laboratory-based study. A cross-over design was utilized in which participants received placebo in one phase and disulfiram (250 mg/d) in the other. Following three days of study medication participants completed two choice sessions. In one they made 10 choices between receiving an intravenous infusion of saline or money that increased in value (US$ 0.05–16) and in the other cocaine (20 mg) or money.

**Results:**

Participants chose cocaine more than saline under both disulfiram and placebo conditions (p<0.05). Unexpectedly, disulfiram increased both the number of cocaine and saline infusion choices (p<0.05). We next examined the relationship between disulfiram dose and cocaine choices. Disulfiram dose (mg/kg bodyweight) was negatively correlated with number of choices for cocaine (p<0.05). Disulfiram also enhanced cocaine-induced increases in cardiovascular measures (p's<0.05–0.01).

**Conclusions:**

Disulfiram's impact on the reinforcing effects of cocaine depends on dose relative to body weight. Our results suggest that the use of weight-based medication doses would produce more reliable effects, consistent with weight-based dosing used in pediatrics and in preclinical research.

**Trial Registration:**

Clinicaltrials.gov NCT00729300

## Introduction

Cocaine dependence continues to be a serious public health problem. The National Survey on Drug Use and Health statistics indicated that the number of current cocaine users approached 1.6 million in 2009 [1]. Despite decades of significant advances in the understanding of the actions of cocaine on neural chemistry, an effective medication treatment for this substance use disorder has remained elusive [2]. Disulfiram (Antabuse) is a medication presently indicated for the treatment of alcohol dependence that has shown potential as a treatment for cocaine dependence in most randomized clinical trials. Disulfiram has several potential mechanisms of action. Among these, disulfiram's metabolite, diethyldithiocarbamate, chelates copper and thereby inhibits many copper-dependent enzymes, including dopamine β-hydroxylase (DβH). This enzyme catalyzes the conversion of dopamine (DA) to norepinephrine (NE). Inhibition of DβH increases brain levels of DA and decreases the synthesis of NE in animals and humans [3]–[8].

Disulfiram treatment also inhibits aldehyde dehydrogenase (ALDH) by a non copper-dependent mechanism [9]. Alcohol is normally metabolized to acetaldehyde, which ALDH metabolizes to acetic acid. ALDH inhibition leads to the buildup of high levels of acetaldehyde after alcohol consumption causing the flushing, nausea, and hypotension characterizing the disulfiram-alcohol reaction. This reaction (or fear of this reaction) is thought to be the mechanism responsible for disulfiram's efficacy in the treatment of alcohol dependence [9]–[11]. In addition, disulfiram inhibits carboxylesterase and cholinesterase by unknown mechanisms [12]–[14]. This interferes with the metabolism of cocaine, increasing plasma levels [15], [16] which may potentiate its cardiovascular effects [17], [18].

In addition to its well known inhibition of the dopamine transporter (DAT) [19], [20] cocaine also inhibits the norepinephrine and serotonin transporters (NET and SERT), increasing synaptic levels of all three neurotransmitters. Cocaine-induced increases in synaptic DA are thought to account for its potent reinforcing effects, though recent research suggests that NE also plays an important role [21], [22].

Clinical studies evaluating the impact of disulfiram treatment have produced divergent results. For example, human laboratory studies have shown that disulfiram treatment decreases cocaine's positive subjective effects [23], increases some of its negative effects such as anxiety and paranoia [24], or produces no changes [17]. Randomized clinical trials of disulfiram as a treatment for cocaine dependence have also been inconsistent. Most studies have found that treatment with disulfiram (250 mg/d) decreases cocaine use [25]–[31] though one well-controlled study [32] found that treatment with lower doses of disulfiram (62.5 and 125 mg/d) increased cocaine use significantly. One study found that disulfiram treatment (250 mg/d) had no effect on cocaine use [33], though participants in that study were poorly compliant, with relatively few taking at least 80% of their prescribed medication doses. Although methodological differences between studies may contribute to these discrepancies, these widely divergent effects of disulfiram need clarification.

The present study was undertaken to help elucidate disulfiram's influence on the reinforcing effects of cocaine in non-treatment seeking cocaine-dependent volunteers. We employed a choice paradigm based on contingency management principles in which participants made a series of choices between receiving doses of cocaine and receiving monetary alternatives of increasing value. As a control, participants also made a series of choices between receiving doses of saline and receiving identical monetary alternatives [34]–[36]. Laboratory studies have demonstrated that concurrently available non-drug alternative reinforcers such as money can decrease choices to self-administer cocaine [35]–[37]. If disulfiram treatment decreased cocaine's reinforcing efficacy then preference for cocaine would decrease and choices for money would increase. Therefore, we hypothesized that disulfiram treatment (250 mg/day) would reduce choices to self-administer cocaine and increase choices for money. Because results from clinical trials suggested that the effects of disulfiram may be dose related, we examined the relationship between disulfiram dose, expressed as 250 mg/kg body weight, and choices for cocaine. We hypothesized that disulfiram treatment would reduce the reinforcing effects of cocaine, indexed as the number of choices for cocaine over money.

## Materials and Methods

The protocol for this trial and supporting CONSORT checklist are available as supporting information; see Checklist S1 and Protocol S1.

### Participants

Seventeen non-treatment seeking cocaine-dependent participants completed the study at the Baylor College of Medicine (BCM) and the Michael E. DeBakey Veteran's Administration Medical Center (MEDVAMC) between February 2009 and August 2009. Participants were recruited using advertisements and paid $500 for completing the entire study. Participants also had the opportunity to earn an additional $104.80 by participating in choice procedures (see below).

All participants met DSM-IV-TR criteria for cocaine-dependence determined using the MINI neuropsychiatric interview. Additional inclusion criteria included age between 18–55 years, a history of cocaine use by smoking or intravenous routes, being in good health as confirmed by a health and physical performed by a physician and having normal routine clinical laboratory blood chemistry tests. Females tested negative for pregnancy by urinary human chorionic gonadotropin. Participants were excluded if criteria were met for dependence on other drugs except for nicotine and cannabis. Additional exclusion criteria included a history of seizures, head trauma, experiencing an adverse effect associated with cocaine use and the presence of other axis I psychiatric disorders. Serious current or chronic medical conditions such as symptomatic HIV disease, asthma, heart and neurological disease were also exclusionary criteria. Use of other centrally acting medications other than disulfiram was not allowed. All participants gave informed consent after being fully informed of potential risks of participating in the study. The Baylor College of Medicine institutional review board approved this study.

### Medications

Sterile cocaine HCl for human use was provided by a contractor for NIDA's Drug Supply Program (RTI International, NC). Sterile cocaine and saline solutions used in the IV self-administration studies were prepared by the MEDVAMC Research Pharmacy. Disulfiram and matching placebo were purchased commercially (Greenpark, Houston, TX). The dose and treatment of disulfiram (250 mg/day) was chosen based on previous cocaine interaction studies showing that treatment with this dose significantly altered the subjective effects of cocaine [23], [24]. This dose was also associated with reductions in cocaine use in clinical trials [25]–[31].

### Study design

Following screening, those that met inclusion/exclusion criteria were housed on the Research Commons of the MEDVAMC for the duration of the study phases. Daily urinary drug screens were employed to ensure compliance. This study was conducted using a double-blind placebo controlled, randomized cross-over design completed in two phases separated by a washout period lasting at least two weeks. In one phase, participants received placebo and in the other phase they received disulfiram, with the order counterbalanced across participants (Table 1). Participants received daily oral doses of disulfiram (250 mg, PO) or placebo from day 2 through day 4 of each study phase (2). Participants completed two choice sessions (one in the AM and one in the PM) on day 4. In one session saline (a control) was available and in the other cocaine was available. Participants made 10 choices between receiving an IV infusion and a monetary alternative, with 15 minutes separating each choice. The value of the money alternative for each of the 10 choices was $0.05, $0.05, $0.05, $0.05, $1, $4, $7, $10, $13, and $16. Money choice amounts ($0.05–16) increased non-linearly in order to engender some cocaine choices when only small amounts of money were available but increased steeply so as to limit the number of participants who made cocaine choices exclusively. The values were selected based on previous experiments [38]. Thus, in each session a participant could earn $51.20 if all money choices were taken or a participant could receive 10 IV infusions if no money choices were taken. If the participant chose an IV infusion (20 mg cocaine or saline), they were allowed to activate a patient-controlled analgesia (PCA) pump that delivered an IV infusion over 2 minutes. The PCA pump was programmed to lock-out after an infusion to prevent any subsequent infusions for 13 minutes. A physician was available during all infusion sessions and participants were monitored for 4 additional hours after the last infusion to ensure safety. Medication was discontinued on day 4 and participants were discharged on day 5. Participants returned to complete the second phase of the study after at least two weeks of washout.

**Table 1:** Study design.

Study Day	Activity
**Phase 1**	
**1**	Admission to GCRC
**2**	Randomized to medication First dose of disulfiram (250 mg, PO)/placebo
**3**	Disulfiram (250 mg, PO)/placebo
**4**	Disulfiram (250 mg, PO)/placebo Choice session 1 (AM): IV cocaine (20 mg)/saline or one of 10 money alternatives in ascending amounts ($0.05, $0.05, $0.05, $0.05, $1, $4, $7, $10, $13, $16)Choice session 2 (PM, opposite of choice session 1): IV cocaine (20 mg)/saline or one of 10 money alternatives in ascending amounts ($0.05, $0.05, $0.05, $0.05, $1, $4, $7, $10, $13, $16)
**5**	Discharge
**Phase 2**	Readmitted after 2 week washout Administered opposite treatment medication of that of Phase 1 (disulfiram/placebo) Same experimental protocol (as above)

The final sample size (n = 17) allowed detection of a large effect size (d = 0.93), assuming two-tailed significance testing, α set at 0.05, and power (1-β) set at 0.95. The power analysis was conducted using g*power [39].

### Outcome measures

Physiological measures including heart rate (HR), systolic (SBP) and diastolic (DBP) blood pressure, and cardiac rate and rhythm (electrocardiograph, ECG) was assessed at the beginning and at numerous time points throughout the experimental sessions (0, 15, 30, 45, 60, 75, 90, 105, 120, 135 min). Participation in the study was ended if any of the following occurred: 1) clinically significant ECG changes developed, 2) HR >130 bpm, 3) SBP >180 mmHg, 4) DBP >120 mmHg. Other outcome measures included number of cocaine IV infusions and money amounts chosen.

### Data analysis

Data were analyzed using SigmaStat 12.0 (SYSTAT Software Inc., San Jose, CA, USA). Parametric tests were utilized unless data sets did not pass normality (Shapiro-Wilk) and equal variance tests then appropriate non-parametric tests were used. IV infusion and money choice data sets did not pass normality and equal variance tests therefore Friedman repeated measures ANOVA on ranks was used to determine possible significant differences. Significant main effects were followed by post-hoc analysis using Student-Newman-Keuls pair-wise multiple comparison procedure on ranks. Possible cardiovascular effects of disulfiram in combination with cocaine were assessed in participants that chose the first cocaine infusion under both placebo and disulfiram treatment conditions. The cardiovascular (HR, SBP, DBP) data passed normality and equal variance tests therefore were analyzed using a 2×2 repeated measures ANOVA with treatment (placebo and disulfiram) and infusion (baseline and first cocaine choice) as factors. Significant main effects were followed with post-hoc multiple comparison procedures (Bonferroni t-test). All data are presented as mean ± standard error. Potential relationships between weight (in kg), disulfiram dose on a mg/kg basis and number of cocaine infusions were assessed using Pearson's *r*. Statistical significance was set at p<0.05.

## Results

### Demographics

**Figure 1:**
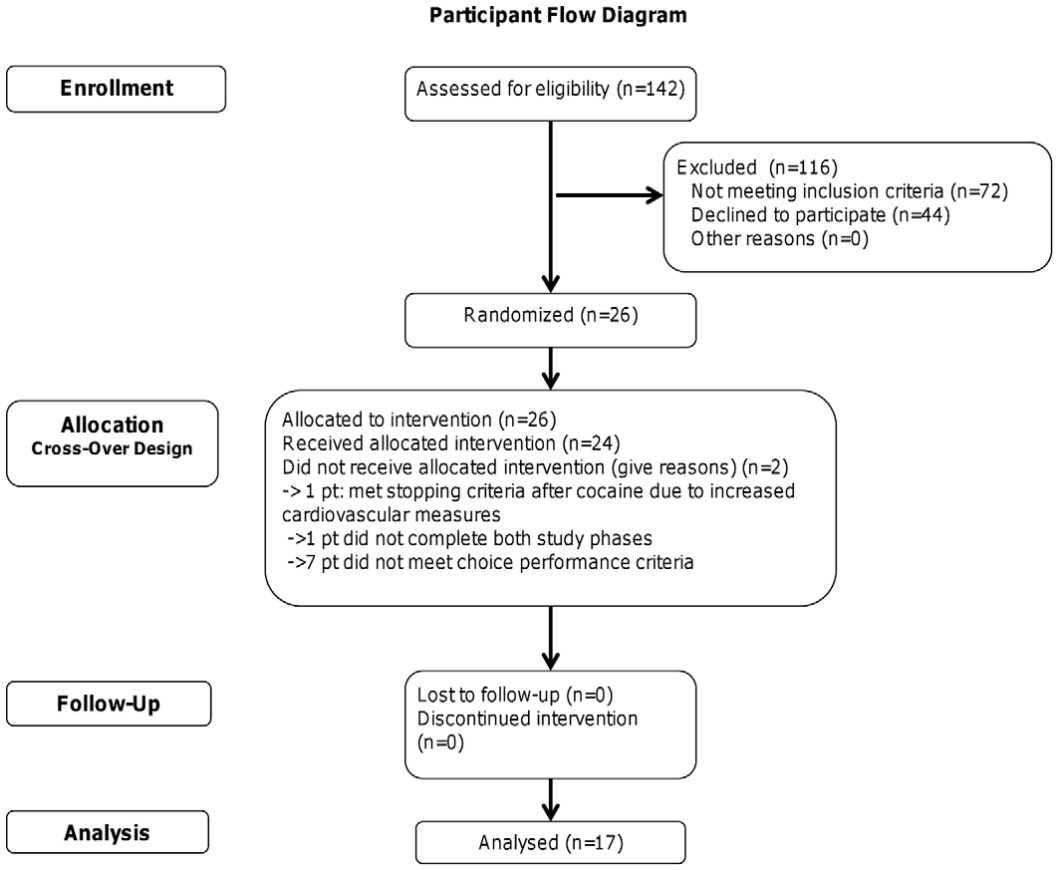
CONSORT Flowchart.

Participant demographics and drug use are presented in Table 2. Twenty-six participants were randomized to receive study medication (see *Participant Flow Diagram*, Figure 1). One did not complete both phases of the study and another was excluded when cardiovascular measures exceeded pre-set stopping criteria. The remaining participants were excluded because they did not choose at least one IV infusion (cocaine or saline) and one money choice in both phases of the study [34], [36], leaving a final sample of 17 participants.

**Table 2:** Participant demographics and drug use.

(N = 17)	
**Gender**	
Male	15
Female	2
**Ethnicity**	
African American	9
Caucasian	7
Hispanic	1
**Age (years)**	41.41±1.61
**Education (years)**	11.88±0.51
**Cocaine**	
Years used	19.00±2.31
Use in last 30 days	18.06±2.25
**Cocaine route of administration**	
Smoke	13
Intranasal	3
Intravenous	1
**Nicotine**	
Years used	14.12±2.72
Use in last 30 days	14.82±2.92
**Alcohol**	
Years used	18.47±2.75
Use in last 30 days	8.47±2.47
**Cannabis**	
Years used	15.24±2.33
Use in last 30 days	3.71±1.89

“Use in last 30 days” indicates number of days of drug use in the 30 days preceding entry into this study.

Participants were middle-aged, mostly African American males, with an average of 20 years of cocaine use. Most used alcohol and cannabis but did not meet criteria for dependence on these substances. A majority of the participants also smoked cigarettes and met criteria for nicotine dependence.

### Drug vs. money choice

**Figure 2:**
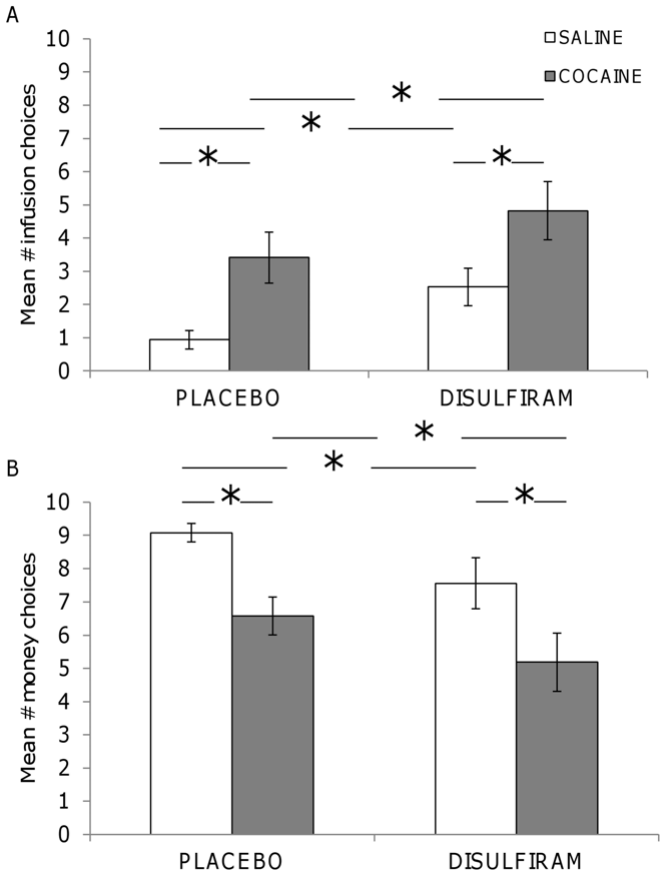
Disulfiram effects on choices in all participants. Impact of disulfiram (250 mg/day) on choices for saline and cocaine (20 mg/IV infusion, A.) and money of escalating value (B.) in non-treatment seeking cocaine-dependent participants (N = 17,* = p<0.05).

Figure 2 presents mean number of IV infusion choices (saline and cocaine, A.) and mean money choices (B.) under placebo and disulfiram experimental conditions across all participants. Analyses showed a significant main effect for infusion choice (*x^2^* = 17.29, p<0.001). Pair-wise multiple comparisons indicated that participants chose more cocaine IV infusions than saline under placebo (p<0.05) and disulfiram (p<0.05) conditions. Participants also chose more saline (p<0.05) and cocaine (p<0.05) IV infusions under the disulfiram condition (p<0.05) compared to placebo. A significant main effect was also observed for numbers of money choices (Figure 2B) [*x^2^* = 17.29, 3, p<0.001]. Post-hoc analysis showed that more money was chosen when IV saline was available under both placebo (p<0.05) and disulfiram (p<0.05) conditions. Significantly less money was chosen by participants under the disulfiram condition when IV saline (p<0.05) and cocaine (p<0.05) was available compared to placebo.

### Cardiovascular effects

Table 3 presents heart rate (HR), systolic blood pressure (SBP), and diastolic blood pressure (DBP) for those participants that self-administered the first available cocaine choice under both placebo and disulfiram treatment conditions (N = 9). Analysis of HR measures revealed a significant main effect for infusion (F_1,35_ = 6.21, p = 0.037). Post-hoc analysis indicated that HR was significantly greater from baseline following a cocaine infusion (p<0.05) under the disufiram but not placebo (p>0.05) condition. Analysis of SBP measures revealed a significant main effect for infusion (F_1,35_ = 16.32, p = 0.004) and a treatment×infusion interaction (F_1,35_ = 9.85, p = 0.014). Post-hoc analysis showed that increases in SBP significantly differed from baseline under the disulfiram (p<0.01) but not placebo (p>0.05) condition. Further, as Table 3 indicates, while treated with disulfiram participants showed greater increases in SBP measures following a cocaine infusion (p<0.05) compared to placebo. A significant main effect for infusion was also found for DBP measures (F_1,35_ = 7.05, p = 0.029). Similar to HR, DBP measures were significantly greater from baseline following a cocaine infusion under the disulfiram (p<0.01) condition but not placebo (p>0.05).

**Table 3:** Cardiovascular effects of disulfiram following a cocaine choice infusion.

*Treatment*	*Heart Rate (bpm)*		*SBP (mmHg)*		*DBP (mmHg)*	
	*Baseline*	*Infusion*	*Baseline*	*Infusion*	*Baseline*	*Infusion*
**Placebo**	77.44±4.8	83.77±5.0	130.33±4.7	135.33±5.0	70.11±3.5	72.55±5.2
**Disulfiram**	76.33±2.4	**85.66±5.8** *	129.11±3.3	**143.11±4.0** * #	72.22±3.0	**79.00±3.9** *

**Data from participants who chose the first cocaine choice under both placebo and disulfiram treatment conditions (N = 9).**

* **significant from respective baseline,**

# **significant from placebo (infusion); bpm = beats per minute; mmHg = millimeters mercury; SBP = systolic blood pressure; DBP = diastolic blood pressure, data presented as mean±SEM.**

### Relationship between disulfiram dose, IV cocaine infusions and money choices

**Figure 3:**
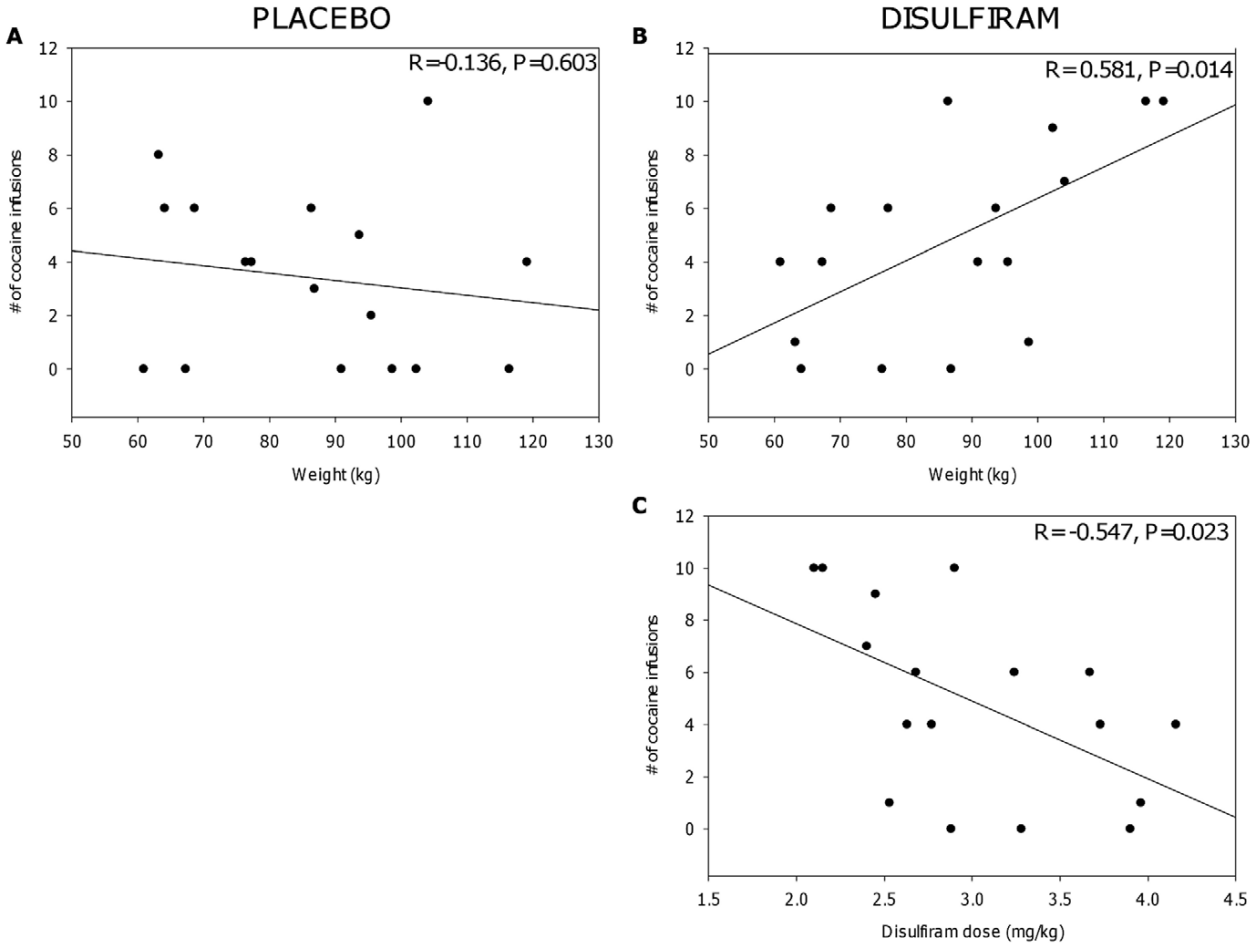
Correlations between weight and number of cocaine infusion choices under placebo and disulfiram treatment conditions. Figure 3A and 3B show the relationship of weight on cocaine choices when treated with placebo and disulfiram. Figure 3C demonstrates the relationship between disulfiram dose calculated on a mg/kg basis and number of cocaine infusion choices under the disulfiram treatment condition. Each data point represents one participant (N = 17).

Although all participants received disulfiram 250 mg/d, their body weights varied nearly 2-fold, ranging from 60 to 119 kg. To take variations in weight into account, we calculated the dose of disulfiram received by each participant on a mg/kg basis. We then calculated the correlation between the number of IV cocaine infusions and the amount of money chosen under the disulfiram condition (Figure 3C). The number of IV choices and value of money choices are presented separately because the value of the money choices increased non-linearly, with later choices worth more money. There was a significant negative correlation between disulfiram dose and the number of IV cocaine choices participants made. That is, as disulfiram dose on a mg/kg basis increased, participants chose fewer cocaine infusions (r = −0.547, P = 0.023). As expected from the foregoing, disulfiram dose was positively correlated with the value of money choices (r = 0.564, P = 0.018, data not shown). As the disulfiram dose increased, participants made more money choices. To ensure this effect was specific to disulfiram, possible relationships were assessed between number of cocaine choices and weight calculated on a kg basis under placebo and disulfiram treatment conditions. As shown in Figure 3A, weight (kg) did not correlate with number of cocaine infusions (r = −0.136, P = 603) when participants were treated with placebo. In contrast, when treated with disulfiram (Figure 3B), weight (kg) positively correlated number of cocaine infusions (r = 0.581, P = 0.014). That is, heavier participants received lower doses of disulfiram that lead to increased choices for cocaine.

## Discussion

We found that disulfiram treatment enhanced cocaine-induced increases in heart rate and systolic blood pressure which is consistent with results from one prior study [17] but not others [23], [24]. Inconsistencies between studies may be due route of cocaine administration and disulfiram dose. The ability of disulfiram to inhibit the metabolism of cocaine thereby increasing peripheral levels may also have contributed to increased cardiovascular effects seen in the present study. Nevertheless, it is clear that disulfiram can enhance cocaine's cardiovascular effects under certain experimental conditions.

This study produced a number of novel and unexpected findings. First, the reinforcing effects of cocaine (the number of IV cocaine infusion choices) was inversely correlated with disulfiram dose calculated on a mg/kg basis. Second, across all participants, disulfiram treatment significantly increased choices for both saline and cocaine and decreased the amount of money choices compared to placebo. Third, disulfiram's effects on choices for cocaine and money were dependent on participants' weight. That is, heavy and light individuals responded similarly to cocaine during treatment with placebo, indicating that weight-related variations per unit dose of cocaine did not play a role in producing the observed outcomes. Weight did however correlate with number of cocaine infusions when treated with disulfiram. This supports the notion that dose on a mg/kg basis is critical to the effects of disulfiram on choice for cocaine.

The finding that disulfiram treatment (across all participants) tended to increase the number of cocaine infusion choices is consistent with another interaction study that showed disulfiram (250 mg/day for 4 days) increased the positive subjective effects of the psychostimulant d-amphetamine (20 mg/70 kg) [40]. This appears to be at odds with outpatient clinical trials showing disulfiram decreases cocaine use [25]–[31]. Our data suggest that treatment with lower doses of disulfiram increased cocaine's reinforcing effects. The effect of dose is better appreciated when participants were assessed based on weight (Figure 2). For example, heavier participants that received a lower dose of disulfiram on a mg/kg basis chose significantly *more* IV cocaine compared to placebo whereas the lighter weight participants that received a higher disulfiram dose did not. Consistent with this, a recent 14 week clinical trial assessing three different doses of disulfiram in cocaine and opioid-dependent volunteers found lower doses of disulfiram (62.5 and 125 mg/day) *increased* cocaine use as measured by urine drug screens whereas the 250 mg dose decreased cocaine use over time [32]. These results are consistent with the finding in the present study that disulfiram's efficacy for reducing the reinforcing effects of cocaine are dose dependent. The mechanism by which low-dose disulfiram treatment enhances the reinforcing effects of cocaine is unknown.

One potential explanation for our finding that on average disulfiram enhanced cocaine's effects whereas most clinical trials found that the same dose (250 mg/d) of disulfiram reduced cocaine use is that we treated participants for several days whereas clinical trials typically include many weeks of treatment. Brief treatment may produce effects similar to low-dose treatment, and chronic treatment or high-dose treatment with disulfiram may therefore be required to reduce the reinforcing effects of cocaine.

Disulfiram is known to inhibit DβH [5], [6] and this appears to be essential for disulfiram to reduce the effects of cocaine and perhaps to reduce drug-seeking more generally. Indeed, in rats, only doses of disulfiram sufficient to reduce brain NE (100 mg/kg) block cocaine-induced reinstatement of cocaine-seeking behavior whereas lower doses that do not affect brain NE (10 mg/kg) do not affect reinstatement [41]. Although we tested only one dose of disulfiram (250 mg/d), our results do suggest that disulfiram's effects are dose-dependent.

DβH mediates the synthesis of NE from DA. Accumulating evidence supports the notion that central NE is necessary for the expression of stimulant-induced effects [2], [21]. Indeed, we recently reported that treatment with doxazosin, a NE α1 receptor antagonist, significantly attenuated cocaine's positive subjective effects in cocaine-dependent volunteers [22].

Unexpectedly, disulfiram increased the number choices for saline infusions, which could be interpreted as an increase in “cocaine-seeking” behavior. These results are consistent with one animal study showing that only doses of disulfiram great enough to decrease DβH—as measured by decreased amounts of central NE—blocked drug-primed reinstatement of cocaine seeking [42]. This interpretation is consistent with the observation that lower doses of disulfiram (62.5 mg and 125 mg/day) increased cocaine use and, by extension, cocaine-seeking behavior [32]. Our study only assessed objective choice behavior and not subjective effects like craving so we lack definitive evidence to support this interpretation. Nevertheless, consistent with the present study, evidence appears to indicate that appropriate doses of disulfiram are needed to decrease the reinforcing effects of cocaine.

Our results indicate that participants treated with approximately 4 mg/kg disulfiram self-administered the least cocaine, whereas participants treated with approximately 2 mg/kg self-administered the most. Our participants weighed on average 86 kg. This indicates that our average participant would require treatment with nearly 350 mg/day of disulfiram to decrease the reinforcing effects of cocaine, at least during short-term treatment. Variations in weight may account for the variable results from clinical trials evaluating disulfiram as a treatment of cocaine dependence, most of which used a fixed dose of 250 mg/day [43]. Studies enrolling low-weight participants would be more likely to find that disulfiram treatment was effective for reducing cocaine use. Unfortunately none of the published studies reported their participants' weight.

Though DβH inhibition can account for consistent findings that higher doses of disulfiram reduce the reinforcing effects of cocaine, we do not have a clear explanation as to how treatment with low-dose disulfiram increases the reinforcing effects of cocaine. Disulfiram's pharmacokinetic and pharmacodynamic effects are complex [9], [44], and other unknown mechanisms are likely involved [45]. For example, an earlier preclinical study found that disulfiram treatment increased cocaine's locomotor activating effects, essentially “pre-sensitizing” rodents to the effects of cocaine [46]. *Dbh* knockout mice are also more sensitive to the effects of cocaine than their wild-type counterparts [47]. An unknown action of disulfiram may account for these unexplained but oddly parallel findings. In humans, it appears that at higher disulfiram doses, dose-dependent inhibition of DβH overcomes this other unknown action of disulfiram.

There are several limitations to this study. First, we focused on behavioral effects and did not obtain subjective measures (i.e. euphoria and craving). This could have contributed to our understanding of how disulfiram enhanced cocaine's reinforcing effects. Second, we administered cocaine by the IV route whereas a majority of our participants took cocaine by smoking, and this may have influenced certain outcomes. Indeed, there is some evidence that the effects of medications may differ depending on whether cocaine is administered by IV or smoked routes [48], [49]. Finally, we treated participants for only a few days, whereas clinical trials last much longer.

In summary, disulfiram treatment was associated with divergent effects on choice for IV cocaine over a monetary alternative that was dependent upon dose on a mg/kg basis. Results suggest that a disulfiram dose of approximately 4 mg/kg most effectively reduced the reinforcing effects of cocaine relative to money and that dose may be an optimal target dose clinically. More extensive studies are needed to clarify the potential therapeutic efficacy of other inhibitors of DβH for cocaine dependence. These results also suggest that interactions studies assessing possible pharmacotherapies for drug dependence administer medications on a mg/kg basis as is done in other areas of research (animal studies and pediatrics) to better reveal possible divergent dose-dependent effects seen with disulfiram.

## Supporting Information

Checklist S1 **CONSORT Checklist.**
(DOC)

Protocol S1 **Trial Protocol.**
(DOC)
