# Supplementary material for: The Impact of Disulfiram Treatment on the Reinforcing Effects of Cocaine: A Randomized Clinical Trial
Source: PLoS One. 2012 Nov 8;7(11):e47702. doi: 10.1371/journal.pone.0047702 (PMC3493584; doi:10.1371/journal.pone.0047702)
Supplement: Protocol S1 — Trial Protocol. (DOC) [file pone.0047702.s002.doc]

**LABORATORY MODELS OF COCAINE SELF ADMINISTRATION**

**Study Design Overview**

We propose to investigate effects of disulfiram 250mg compared to placebo on cocaine self-administration using a human laboratory model established by a group at Johns Hopkins (Dr. Bigelow). This investigator has agreed to serve as a consultant for this project, ensuring that the procedures followed are appropriate and that interpretation of the data is consistent with prior studies. Letters from the consultants are included in the appendix

Participants will be admitted to the hospital for 2 study visits. The visits follow the same layout, and participants will receive study drug during one visit and placebo during the other. Participants will be admitted to the hospital and will be randomized to study drug or placebo on the following day. During the next several days staff will familiarize participants with upcoming procedures and participants will complete a mock version study procedures. Participants will remain seated during experimental sessions except for brief visits to the lavatory. The first 30-min of all sessions will be devoted to the collection of baseline data and participant preparation.

At 1pm on days 5 and 7, participants will choose between a dose of cocaine (0mg or 20mg) or a monetary alternative in each of 10 subsequent choices. The same dose of cocaine, 0mg or 20mg, will be available for all 10 choices on a given day and each dose will be assigned to either day 5 or 7 in a randomized, double-blind manner. The monetary alternative will be $.05, $.05, $.05, $.05,$1, $4, $7, $10, $13, or $16 cash, presented in that order. Selections will be made at 15-minute intervals, and participants will receive cocaine doses or cash immediately after indicating their choice, providing vital signs remain within preset intervals. Heart rate variability measures will be conducted throughout the choice session as well.

Study drug will be discontinued on day 7, after completion of the experimental sessions. On day 8, the neurocognitive assessment and continuous heart rate recording will be repeated (as on days 1 and 4), starting at 3:00 PM. Again, subjects will be asked to refrain from smoking tobacco for at least 2 hours before the assessment.

Subjects will be monitored through day 8 and on day 9 they can either go home and return to complete the procedures under the alternate condition (placebo vs. disulfiram), or they can stay in the hospital and start the alternate study drug on the next day.

Subjects can be re-admitted to the study after several days or weeks and the experiment will be repeated during treatment with the alternate study drug (placebo or disulfiram). The total possible dose of cocaine that a patient can receive during choice sessions is 200mg, and the total dose they can receive over the study visit is 200mg.

In both studies, the participants will respond by pressing a response button or lever, depending on whether the patient controlled analgesia (PCA) pump or the Med Associates device incorporating FDA-approved Baxter infusion pump is utilized. When money choices have been selected, cocaine choices are unavailable for the remainder to the time-out period. For the purpose of these studies, the devices are interchangeable.

**Research Participants**

Each subject will complete procedures while receiving placebo and disulfiram. We anticipate that 75 volunteers will enter the study and that there will be a dropout rate of 20-30%, yielding a sample size of 60.

*Recruitment*

Potential participants will be recruited using flyers, print ads, and radio advertisements. Our experience from previous studies indicates that well-placed and maintained flyers coupled with print and radio spots are sufficient to maintain frequent calls to our toll-free number. Over a recent 12-month period, our recruitment center received approximately 2,600 calls from stimulant users interested in participating in our studies. From those contacts, approximately 10% of the callers were eligible to meet with our research staff for consultation. Roughly 20% of those callers attended an initial screening appointment and met general inclusion criteria for a non-treatment-seeking study involving cocaine or methamphetamine administration (fewer met stricter criteria for particular studies, as some studies require prior IV experience). Thus, our existing recruitment approach provided about 50 eligible participants per year. We can easily increase the intensity of recruitment efforts in to accommodate the recruitment needs of the proposed project.

Participants who call our toll-free number are informed about the *general* requirements of the study and appointments are scheduled for those who are interested. Participants are not informed about the specific inclusion/exclusion criteria in order to avoid participants tailoring their responses in order to enter the protocol. During a face-to-face interview with a study physician participants are given consent documents that are read to them if needed. Following completion of the informed consent process, participants are screened using the criteria below. At several times during the screening process, participants are informed that treatment is available and referrals are provided if desired. Following completion of the consent process, participants will an oral swab will be used to collect epithelial cells that will provide DNA for genotyping at the DBH locus. Subjects with all genotypes will be included in this study.

*HIV issues.* After informed consent is obtained (described in *Human Subjects*), each participant will undergo HIV testing during their medical screening. A separate consent form for HIV testing provided by UCLA is signed and maintained in the patient’s clinical chart. In the event that any participant tests positive for HIV, he/she will be provided with counseling and medical follow-up. HIV itself is not exclusionary, though the presence of active disease is. Current use of antiretroviral medication is also exclusionary.

*Gender and race/ethnicity.* Participants will be selected without regard to gender or race/ethnicity. The estimates provided are based on our previous experience recruiting research volunteers. Due to the under-representation of women in drug abuse research, we will maximize recruitment of women by targeted advertising. Admission of women will be timed so that cocaine administration occurs during the follicular phase of the menstrual cycle, because cocaine plasma levels are greater then compared to the luteal phase (Lukas et al 1996).

*Participation of children.* Individuals under the age of 21 will not be excluded. Children younger than 18 years old will not be included in the proposed project because cocaine dependence (aside from abuse) is relatively rare in children under 18. The target population for this potential pharmacotherapy is cocaine-dependent adults, so participants for this study will sample from that population (see *Human Subjects*).

*Inclusion Criteria*

In order to participate in the study, participants *must*:

1. Be English-speaking volunteers who are not seeking treatment at the time of the study;
2. Be between 18-55 years of age;
3. Meet DSM IV criteria for cocaine dependence;
4. Have a self-reported history of using cocaine by the smoked or IV route. Participants must report at least weekly use for the past month and have a cocaine-positive urine test in the week of study entry;
5. Have vital signs after a 48 hours washout period as follows: resting pulse between 50 and 90 bpm, blood pressures between 85-150mm Hg systolic and 45-90mm Hg diastolic;
6. Have hematology and chemistry laboratory tests that are within normal (+/- 10%) limits with the following exceptions: a) liver function tests (total bilirubin, ALT, AST, and alkaline phosphatase) < 3 x the upper limit of normal, and b) kidney function tests (creatinine and BUN) < 2 x the upper limit of normal;
7. Have a baseline ECG that demonstrates normal sinus rhythm, normal conduction, and no clinically significant arrhythmias; and
8. Have a medical history and brief physical examination demonstrating no clinically significant contraindications for study participation, in the judgment of the admitting physician and the principal investigator.

*Exclusion Criteria*

Potential participants will be excluded from participation in the study if any of the following apply:

1. Have any history or evidence suggestive of seizures or brain injury
2. Have any previous medically adverse reaction to cocaine, including loss of consciousness, chest pain, or epileptic seizure;
3. Have neurological or psychiatric disorders, such as:

- psychosis, bipolar illness or major depression as assessed by MINI (or SCID if necessary);
- organic brain disease or dementia assessed by clinical interview;
- history of any psychiatric disorder which would require ongoing treatment or which would make study compliance difficult;
- history of suicide attempts within the past three months assessed by MINI and/or current suicidal ideation/plan as assessed by MINI;

1. Have evidence of clinically significant heart disease or hypertension, as determined by the PI;
2. Have a family history in first degree relatives of early cardiovascular morbidity or mortality, as determined by the PI;
3. Have evidence of untreated or unstable medical illness including: neuroendocrine, autoimmune, renal, hepatic, or active infectious disease;
4. Have HIV *and* are currently symptomatic, have a diagnosis of AIDS, or are receiving antiretroviral medication;
5. Be pregnant or nursing. Other females must either be unable to conceive (i.e., surgically sterilized, sterile, or post-menopausal) or be using a reliable form of contraception (e.g., abstinence, birth control pills, intrauterine device, condoms, or spermicide). All females must provide negative pregnancy urine tests before study entry, upon hospital admission, and at the end of study participation;
6. Have any history of asthma, chronic coughing and wheezing, or other respiratory illnesses;
7. Currently use alpha or beta agonists, theophylline, or other sympathomimetics;
8. Currently meet criteria for alcohol dependence or refuse to abstain from alcohol during the protocol and for at least one week after discharge.
9. Have any other illness, condition, or use of medications, which in the opinion of the PI and/or the admitting physician would preclude safe and/or successful completion of the study.

##### *Criteria for Discontinuation Following Initiation*

1. positive urine toxicology or breath test indicating illicit use of cocaine, amphetamine, alcohol, opiates, or other abused drugs not delivered as part of this protocol;
2. inability to comply with study procedures;
3. meet discontinuation criteria due to exaggerated response to cocaine, described below.

*Rationale for Subject Selection Criteria*

Participants are required to have used cocaine by the IV or smoked route to avoid exposing participants to routes of administration that produce more intensive interceptive effects. The age criteria were selected primarily to avoid enrolling participants with undiagnosed cardiovascular disease. Participants with active HIV disease are excluded to avoid potential exacerbation of their underlying disease; participants with asymptomatic HIV are included because this group is at high risk for cocaine dependence. Participants with asthma (or who take asthma medications) are excluded due to potential adverse interactions between beta agonist medications and cocaine. Patients who heavily use alcohol are excluded due to the potential for disulfiram-alcohol reaction in the several days following completion of the protocol.

*Rationale for genetic assessments.* Data provided by Dr. Schottenfild from an ongoing clinical trial shows that disulfiram was superior to placebo on both reported use and positive urine toxicology in patients with the DBH T allele (associated with lower enzyme activity), whereas in patients without the T allele disulfiram was superior to placebo only on reported use measures. Presumably, patients with the T allele have lower baseline DBH activity, and this is more sensitive to inhibition by disulfiram. We elected to allow participants with all DBH genotypes but test for DBH and COMT genotypes and to preferentially include patients with the C/T genotype in an effort to collect data from those most likely to respond to the medication. We will attempt to genotype participants before admission into the GCRC so that beds can preferentially be given to participants with the C/T genotype. Those with the C/C genotype will be enrolled when there is space available.

The infectious disease panel for hepatitis is performed as an aid to determine if the prospective subject has been exposed to a hepatitis virus. Positive hepatitis results do not exclude a prospective subject from participation unless there is an indication of active liver disease. Similarly, a positive tuberculin (PPD) result does not exclude a prospective subject from participation, but if diagnostic tests (e.g. chest x-ray) indicate that active disease is present, subjects may be excluded from participation. If any test results are positive subject will be notified of positive and confirmatory test results by Dr. Newton and will be referred for treatment. If positive, they will be referred for appropriate follow-up and/or treatment. Dr. Newton will provide subjects with counseling and medical follow-up. They will not be charged for the counseling but you will be responsible for the cost of any medical treatment they need as a result of the diagnosis.

*Payment to Research Participants*

Subjects will be paid $25 in gift certificates to local restaurants and stores for the completion of the initial screening. Subjects may have the option of filling out additional assessments at the end of the initial screen, for an additional $20 in gift certificates. Subjects will receive $30 in gift certificates for completion of the medical screening, including interviews, blood tests, and ECG, which may be scheduled over several days. Subjects will be paid $50 per day, with an additional $100 if they complete the entire study. If they drop out of the study or are excluded at some point, they will be paid according to the number of days participated but the payment date will not change. Payment will occur on the date that would have been the completion date, and the follow up dates two and possibly four weeks later regardless of when the patient leaves the study. Exceptions may be made for participants who are excluded after enrolling in the study but did not choose to leave. Subjects will not receive the entire payment at once, but will be paid according to the following schedule:

Completion of part 1: $100 (cash)

Completion of part 2: $300 (cash)

2-week follow up visit: $300 (cash)

4-week follow up visit: $300 (cash)

For subjects completing the entire study, the total will consist of $1055, of which $1000 will be paid in cash and $55 in gift certificates (or $75 in gift certificates if subjects complete the optional additional screening). Subjects are required to fill out a W-9 form for their payment as part of this study.

Subjects will also have the opportunity to receive money as part of the choice sessions. Across the choice sessions that they participate in, they would be able to receive up to a maximum of $204.80. The actual amount subjects receive will be substantially less because no subject chooses money each time. Subjects must spend the cash earnings prior to being discharged from the GCRC. Study staff will accompany subjects to local stores to make purchases.

**Study Drugs and Dosing Protocols**

*Regulatory issues*. An IND exemption for the use of cocaine and disulfiram in this protocol will be obtained from the FDA. NIDA regulatory staff routinely agrees to provide FDA with a letter granting access to NIDA’s master drug file for cocaine for other projects. Disulfiram is an approved drug and the package insert serves as reference to the master drug file. The PI has a DEA certificate allowing prescription of Schedule II drugs, including cocaine.

*Cocaine.* Human use cocaine HCl in 20mg (10mg/ml in 2ml ampoule) and 40mg (20mg/ml in 2ml ampoule) doses will be provided by a NIDA contractor. The compound will be stored in the UCLA Research Pharmacy vault. Cocaine and matched placebo saline will be administered by IV infusion over at least 60 seconds.

*Study medications.* Disulfiram tablets will be purchased commercially and managed by the Research Pharmacy. The pharmacy will encapsulate the tablets and make matched placebo tables. Participants will take one capsule containing 250mg disulfiram or placebo each morning. The order in which participants receive placebo or disulfiram will be randomized.

*Cocaine dose justification.* In our prior laboratory investigations of effects of cocaine, we have utilized individual doses of 40mg given IV. This dose has been selected to safely produce significant cardiovascular and subjective effects, without reaching a ceiling effect. Maximum doses of 20mg will be used in the choice experiments because the dose will be administered repeatedly, and this dose has been shown to be safe in that context.

*Disulfiram dose justification.* Data recently made available by Drs. Oliveto and Kosten (to be presented at the ACNP, 2004) indicate that treatment with low doses (62.5mg or 125mg) was actually associated with increased cocaine use compared to placebo in a clinical trial involving methadone-maintained patients. Further, there were indications that those with higher DBH activity were less responsive to 250mg disulfiram relative to those with lower DBH activity. A second study (Schottenfeld et al., presented at the CPDD, 2004) among patients with a T allele, 250mg disulfiram was superior to placebo on both self-report and urine drug screen results (Carroll et al 2004). We considered using the higher disulfiram dose, based on Oliveto and Kosten’s data showing that participants with lower enzyme activity responded better to disulfiram treatment. Presumably, those with higher enzyme activity would respond to a higher dose of disulfiram. We elected not to include the higher dose, but rather to select those with the T allele, based on the logic that T allele carriers would tend to have levels of enzyme and further, based on Schottenfeld’s data, should respond to the 250mg dose.

*Safety of Cocaine and Disulfiram.* The most important risks associated with cocaine administration alone are related to the sympathomimetic effects of the drug. Other risks include changes in mental status, including the development of anxiety and aversive effects. These may be worsened by combined treatment with disulfiram. The most important risks associated with disulfiram treatment are the disulfiram-ethanol interaction, hepatic toxicity, optic neuritis, peripheral neuritis, polyneuritis, and peripheral neuropathy. These and other risks are discussed in detail in the *Human Subjects* section below.

The risks associated with combined cocaine and disulfiram treatment may be inferred from previous research involving intranasal cocaine (Hameedi et al 1995; McCance-Katz et al 1998a; McCance-Katz et al 1998b). In spite of higher cocaine plasma concentrations, disulfiram treatment was not associated with increased cardiovascular effects, probably due to acute tolerance. In this study, we anticipate that disulfiram will also not be associated with an enhanced cardiovascular response to cocaine, but that disulfiram treatment will be associated with reduced reinforcing effects, which would be consistent with the clinical data.

**Assessments**

*Genetic Assessments*

*DNA Collection/Isolation.* We will collect DNA from buccal swabs applied to Whatman FTA cards. These cards allow safe and stable storage of biological samples for DNA extraction. Our anticipated yield of genomic DNA is 50-100µg which is adequate for over 500 genotype assays using currently available methods (Del Rio et al 1996; Karle 2001).

*Genotyping.* Genotypes will be determined using 5’ Exonuclease-based (Taqman) genotyping assays. Assays will be developed by Applied Biosystems (ABI; Assays by Design). Allele discrimination will be performed using the ABI 3730 realtime PCR cycler (Geller et al 2004). These procedures will be conducted by the Co-Investigator Dr. Charles Glatt at UCLA.

*Rationale for genetic assessments*. We elected to test participants for the DBH C/T genotype based on data provided by Dr. Schottenfild from an ongoing clinical trial showing that disulfiram was superior to placebo on both reported use and positive urine toxicology in patients with the DBH T allele (associated with lower enzyme activity), whereas in patients without the T allele disulfiram was superior to placebo only on reported use measures. Presumably, patients with the T allele have lower baseline DBH activity, and this is more sensitive to inhibition by disulfiram. We elected to test participants for genotype rather than DBH activity, though the latter is probably more relevant, for two reasons. First, DBH activity is found as a continuum in the population and arbitrary cut-offs would be needed.. Second, the assay for DBH activity is more expensive and less easily performed than the genotyping procedures (at least for our Center).

*Clinical Assessments*

*Addiction Severity Index (ASI)- Lite CF Version*. The ASI-Lite CF version (McLellan et al 1992; McLellan et al 1988) will be administered by a trained research staff member. The ASI-Lite is the interviewer’s estimate of the severity of the participant’s status in seven areas (medical, employment, drug use, alcohol use, legal, family/social, and psychological). The Lite version is a shorter version of the ASI that still retains all questions used to calculate the ASI composite scores. We plan to retain the family history section of the ASI, as the ASI–Lite version collects minimal family history information.

*Substance Use Inventory (SUI).* This instrument collects data on the type, frequency, and amounts of drugs used, as well as routes of administration. Participants will complete this measure during screening to establish severity of drug use for the prior six weeks and at intake to assess drug use during the interval between intake and the time that the SUI was completed during screening.

*Structured Clinical Interview for the DSM-IV (SCID)*. This instrument will be administered during intake screening and serves to determine whether the participant meets the DSM-IV criteria for drug dependence and to rule out any major psychiatric disorders (e.g., affective disorders, schizophrenia) (Spitzer 1995).

*Beck Depression Inventory (BDI).* The BDI is a 21-item self-report inventory that focuses on the participant's subjective feelings of depression and is sensitive to changes over time (Beck et al 1968). Participants will complete the measure during intake screening.

*Medical history and physical exam.* Personal and family health profiles and medical history will be collected prior to the examination and reviewed with a licensed medical professional. A physical exam, neurologic exam, and neuropsychiatric mental status evaluation will be conducted.

*Medical/Pharmacological Assessments*

*Cardiovascular indices*. Beginning 15 minutes before cocaine administration and at 5 to 10 minute intervals thoughout the cocaine administration session, continuing until 240 minutes after the final cocaine administration, blood pressure and heart rate will be monitored using automatic heart rate and blood pressure assessment.

*Clinical laboratory assessment*. Blood for hematology studies will be collected in anticoagulant-containing vacutainer tubes for analysis of CBC, chemistry, liver function tests, and renal function tests. HIV assessment will consist of ELISA with Western-blot confirmation of positive results. A urine-based pregnancy test for females will measure human chorionic gonodotropin.

*Cocaine self-administration procedures.* The participant will indicate the selected option, cocaine or money. For cocaine administration participants will push a button on a PCA pump and will receive the dose immediately.. For delivery of money choices, the research staff will visibly put the money in an envelope designated for the patient for use later on.. Cardiovascular and subjective effects will be assessed prior to and at 5, and 14 minutes after each dose of cocaine. If cardiovascular measures have not returned to acceptable levels (heart rate <130 bpm, diastolic pressure <100mmHg, and systolic pressure <165mmHg), the next choice session will be delayed by 15 minutes, after which these measures will be repeated. These procedures will be repeated for each cocaine self-administration session.

*Cardiovascular monitoring and adverse event management.* We have developed standard operating procedures aimed at allowing safe administration of cocaine in a medical setting. These have been successful, evidenced by our completed and ongoing studies. Participants will be monitored in an ICU-like setting at the Medical Center. An ACLS-certified physician will perform all cocaine administrations and will be present until vital signs have returned to normal. Specific guidelines have been developed to guide assessment and intervention in the event of cardiovascular toxicity, such as malignant hypertension, ventricular arrhythmia, chest pain, etc., and are included in the Appendix. These are intended to support rather than replace clinical judgment. If needed, a “code team” responds. Patients who need to be transferred to a medical or ICU ward can be, though this has never been necessary.

*Stopping Criteria*

Participants must continue to meet inclusion criteria in order to remain in the protocol *(*see criteria for discontinuation following initiation, above.). Thus, cocaine administration will not be initiated if vital signs are outside of acceptable ranges: resting pulse between 50 and 90bpm and blood pressure below 150mm Hg systolic and 90mm Hg diastolic. Repeated doses of cocaine will not be administered (and the study physician will halt continued cocaine delivery by the syringe pump) if any of the following pertain: HR > 130bpm; Diastolic BP > 100mm; Systolic BP > 165mm; Behavioral manifestation of cocaine toxicity (agitation, psychosis, inability to cooperate with study procedures).

*Stopping criteria for further study participation.*  Further participation of the participant is stopped if any of the following events occur: Stopping criteria for further cocaine administration do not return to acceptable limits within 30 minutes; Stopping criteria for further cocaine administration are met for a second time within the protocol; Systolic BP > 180mm Hg sustained for 5 minutes or more; Diastolic BP > 120mm Hg sustained for 5 minutes or more; Heart rate > (220 – age x 0.85) bpm sustained for 5 minutes or more. These are extreme values and we do not anticipate this would occur based on prior experience by ours and other groups administering repeated doses of cocaine in the laboratory.

**Protocols and Procedures**

Participants will reside as inpatients in the Clinical Research Center for the duration of this study. Participants will be required to refrain from illicit and prescription drug use for the duration of the study and this will be confirmed with daily urine and breath alcohol level testing. Caffeine and food will be prohibited during and 1-2 hours before study sessions.

Experimental sessions will be conducted at approximately the same time of day for a given participant. Experimental sessions will be several hours in duration and will be conducted in a quiet room on the GCRC with research and medical staff present. The room will contain an experimental station, equipped with a response console attached to a personal computer and video monitor. Participants will register selections by pressing a key on the response console. Heart rate, EKG, and blood pressure will be recorded throughout experimental sessions using a Scout automatic monitoring system. Medical personnel will monitor cardiac function during drug administration periods and for 2 hours following drug administration.

Participants will be admitted to the hospital and will be randomized to study drug or placebo on the following day. During the next several days staff will familiarize participants with upcoming procedures and participants will complete a mock version study procedures. Participants will remain seated during experimental sessions except for brief visits to the lavatory. The first 30-min of all sessions will be devoted to the collection of baseline data and participant preparation.

**Experiments**

We propose assess effects of disulfiram and placebo on cocaine self-administration using an established laboratory model. Each participant will complete the experiment during treatment with placebo and disulfiram. The order in which a participant will receive disulfiram or placebo will be randomized to control for order effects.

At 1pm on days 5 and 7, participants will choose between a dose of cocaine (0mg or 20mg) or a monetary alternative in each of 10 subsequent choices. The same dose of cocaine, 0mg or 20mg, will be available for all 10 choices on a given day and each dose will be assigned to either day 5 or 7 in a randomized, double-blind manner. The monetary alternative will be $.05, $.05, $.05, $.05,$1, $4, $7, $10, $13, or $16 cash, presented in that order. Selections will be made at 15-minute intervals, and participants will receive cocaine doses or cash immediately after indicating their choice, providing vital signs remain within preset intervals. Heart rate variability measures will be conducted throughout the choice session as well.

Study drug will be discontinued on day 7, after completion of the experimental sessions. On days 1, 4 and 8, neurocognitive assessments and continous heart rate recording will be completed starting at 3:00 PM. Subjects will be asked to refrain from smoking tobacco for at least 2 hours before the assessment.

Subjects will be monitored through day 8 and on day 9 they can either go home and return to complete the procedures under the alternate condition (placebo vs. disulfiram), or they can stay in the hospital and start the alternate study drug on the next day.

Day 1 Admit to the CRC, complete neurocognitive testing with continuous heart rate recording.

Day 2 *Randomize to study drug 1. The order in*

which participants *receive disulfiram*

*and placebo will be randomized*

Day 3

Day 4 Neurocognitive testing with continuous heart rate recording.

Day 5 Choice session 1

*Participants will choose between cocaine (0mg or 20mg) and money*

Day 6

Day 7 Choice session 2

*Participants will choose between cocaine (0mg or 20mg, reverse of day 5) and money*

Day 8 Monitor. Neurocognitive assessment with continuous heart rate recording.

Day 9 Discharge or subjects can remain in hospital for study part 2

The total possible dose that a patient can receive during choice sessions is 200mg, and the total dose they can receive over the study visit is 200mg.

A participant’s day will begin at 7 am with study drug dosing followed by breakfast. Lunch will be served at 11:30. At 1 pm participants will enter the choice component of the experiment. This will be completed by 5 pm, after which dinner will follow at 6 pm. The dependent measures will be (1) the maximal completed ratio attained for cocaine within a session, (2) baseline (prior to the first sample dose) subjective effects (assessed using visual-analogue scales) and cardiovascular indices, (3) effects of the sample dose of cocaine on subjective effects, cardiovascular, and performance measures, and (4) effects of repeated cocaine doses on subjective effects and cardiovascular measures using time as a within-group factor (relevant to sessions in which cocaine is selected). The planned comparisons for each measure are disulfiram versus placebo at each dose of cocaine (Haney et al 1998).

*Follow-up of Study Participant*s

Participants will return to the study site at 2 weeks and at 4 weeks following completion of each study. Adverse event, drug use, and clinical information will be completed, and participants will receive compensation for participating in the study.

**Data Collection and Management**

*Staff training.* Staff will be trained to collect data utilizing good clinical practices, and monitors will ensure that that standard is adhered to. For assessments completed by two different individuals, mock assessments will be used to ensure that there is adequate agreement (the specific kappa level depends on the assessment).

*Data collection.* Data will be collected using paper-and-pen or direct computer entry and transcribed onto teleform data sheets. A trained research assistant will perform quality assurance monitoring of each subject’s file to assure that there are no variances in collection procedures over time and to assure completeness.

**Statistical Analysis**

*Power analysis.* The proposed sample size of 60 should allow detection of small-to-medium effects (approximately f=.17) when comparing conditions (within-subject factors) using repeated measures analyses with power=.80 and two-tailed alpha=.05. This effect size translates to a difference of approximately 1/3 standard deviation units (d=.33) in differences in means across conditions (Cohen 1988; Elashoff 2000). A moderate within-subject correlation of .5 was assumed across conditions. We have conservatively powered the study for 2-tailed tests to cover the range of primary and secondary hypotheses; however, since we expect disulfiram to reduce cocaine choices, power would be greater for a one-tailed test of the primary hypothesis in each experiment. This sample size will also allow for detecting medium-to-large effects in assessing secondary hypotheses (such as a linear trend in cocaine dose-response) with Bonferroni correction for post hoc comparisons. The above power analysis has been calculated assuming no order effects for disulfiram vs. placebo conditions; however, if order effects are detected, then analyses will assess effects within order conditions, with a corresponding decrease in power allowing detection of medium effect sizes (f=.26) across condition.

*Statistical Analysis.* Descriptive statistics will be used to summarize sample characteristics on all measures, as appropriate to measurement characteristics of individual variables. Order effects (for disulfiram vs placebo) will be assessed using repeated measures ANOVA to determine equivalence; because of random assignment, no differences are anticipated. However, if significant differences are found, then primary analyses will examine effects within each order condition.

Repeated measures ANOVA will be used to assess effects of disulfiram within each experiment. For each experiment, a general linear model will include within-subject factors representing condition (disulfiram vs. placebo) and cocaine dosage. Separate analyses will be conducted for each outcome measure. If outcome measures are substantially non-normal, then a GEE (generalized estimating equations) approach will be used for testing hypotheses instead of the general linear model for repeated measures (Burton et al 1998). This extended approach allows analysis of non-normally distributed data (e.g. binomial or gamma) as well as inclusion of a correlated covariance structure to accommodate within-subject effects.

# Anticipated Potential Difficulties and Solutions

Few cocaine users carrying the DBH C/T genotype may volunteer for the study. This evidence for genetic self-selection would be a critical finding in itself, and would strongly support the use of treatments similar to this one. This is unlikely, as Schottenfeld found that 50 of 128 (40%) of cocaine- and opiate-dependent volunteers carried the DBH T allele. Nevertheless, if needed, we can increase our access to potential subjects through collaboration with our outpatient clinics, where hundreds of additional appropriate participants are available.

Cocaine may produce excessively aversive effects during disulfiram treatment in participants with the DBH T allele, leading to a high rate of study dropout. This would be crucial information for planning subsequent clinical trials. This is unlikely, however, based on Schottenfeld’s data showing that patients carrying the T allele completed his clinical trial. If participants report excessively aversive effects of cocaine, we would restrict the cocaine dose to acceptable levels, thus ensuring the feasibility of this study.

*Recruitment.* Critical to any clinical study is recruitment of sufficient numbers of participants. We believe that we have identified effective advertisement approaches, but in the event that recruitment numbers are not sufficient, we plan to increase our sources of participants by expanding our recruitment activities to ISAP-affiliated clinics treating stimulant abusers in Rancho Cucamonga and Costa Mesa, towns 50 and 30 miles distant from UCLA, respectively. We plan to set up offices separate from the treatment component of the clinic where non treatment-seeking volunteers can be identified and consented. Taxis or airport vans will then be used to transport volunteers to UCLA.

*Retention.* Retention has generally not been problematic. If difficulties were to develop, we would identify the source of the difficulty via interviews with departing participants. If systematic difficulties emerged, we would address them. If the inpatient CRC stay constitutes part of the difficulty a potential solution is outlined in *Bed Space*, below.

*Bed space.* The CRC is a finite resource and competition can emerge from other researchers for bed space. We have anticipated this possibility and have trained psychiatric nurses from the UCLA Neuropsychiatric Hospital to provide nursing assessment and care as part of protocols similar to this one. Trained study staff will collect all research-related assessments. In addition, we have developed a collaboration with staff at Cedars Sinai hospital, located 3 miles from UCLA. This facility has a NIH-supported CRC and our usual staff may assess participants there via a subcontract.
